# EMS Mutagenesis in the Pea Aphid *Acyrthosiphon pisum*

**DOI:** 10.1534/g3.113.009639

**Published:** 2014-02-13

**Authors:** Denis Tagu, Gaël Le Trionnaire, Sylvie Tanguy, Jean-Pierre Gauthier, Jean-René Huynh

**Affiliations:** * UMR 1349 IGEPP (Institute of Genetics Environment and Plant Protection), Domaine de la Motte, 35657 Le Rheu Cedex, France; † Institut Curie, CNRS, Genetics & Developmental Biology, UMR3215,Inserm U934, F-75248 Paris, France

**Keywords:** pea aphid, mutagenesis, EMS, X-linked mutation

## Abstract

In aphids, clonal individuals can show distinct morphologic traits in response to environmental cues. Such phenotypic plasticity cannot be studied with classical genetic model organisms such as *Caenorhabditis elegans* or *Drosophila melanogaster*. The genetic basis of this biological process remain unknown, as mutations affecting this process are not available in aphids. Here, we describe a protocol to treat third-stage larvae with an alkylating mutagen, ethyl methanesulfonate (EMS), to generate random mutations within the *Acyrthosiphon pisum* genome. We found that even low concentrations of EMS were toxic for two genotypes of *A. pisum*. Mutagenesis efficiency was nevertheless assessed by estimating the occurrence of mutational events on the X chromosome. Indeed, any lethal mutation on the X-chromosome would kill males that are haploid on the X so that we used the proportion of males as an estimation of mutagenesis efficacy. We could assess a putative mutation rate of 0.4 per X-chromosome at 10 mM of EMS. We then applied this protocol to perform a small-scale mutagenesis on parthenogenetic individuals, which were screened for defects in their ability to produce sexual individuals in response to photoperiod shortening. We found one mutant line showing a reproducible altered photoperiodic response with a reduced production of males and the appearance of aberrant winged males (wing atrophy, alteration of legs morphology). This mutation appeared to be stable because it could be transmitted over several generations of parthenogenetic individuals. To our knowledge, this study represents the first example of an EMS-generated aphid mutant.

The possibility to perform forward genetic screens has significantly contributed to the success of model organisms such as *Drosophila melanogaster*, *Caenorhabditis elegans*, or *Arabidopsis thaliana* (Jorgensen and Mango 2002; Page and Grossniklaus 2002; Patton and Zon 2001; St Johnston 2002). One of the main advantages of this approach is that very few starting hypothesis need to be made. It is only assumed that the process of interest is based on information contained in DNA sequences and that can be altered by mutagens. This strategy is thus not hypothesis-driven and allows deciphering truly novel and unexpected mechanisms. Furthermore, large-scale screens can aim at saturating the genome and help discovering entire signaling or genetic pathways. A widely used chemical mutagen is the alkylating agent ethyl methansulfonate (EMS), which mostly induces GC to AT point mutations. A large number of reports show that EMS-based screens are truly random and can cover the entire genome, show a high mutation rate, and produce a wide variety of alleles, such as protein-null, hypomorphic, conditional, or domain-specific mutations (Ashburner *et al.* 2005). On the down side, this approach is very labor-intensive and requires the rearing and handling of large numbers of individuals. Another important bottleneck is the mapping and molecular identification of EMS-induced DNA lesions. These two disadvantages have restricted the use of forward genetic screens to a limited number of model organisms with a small body size and a short generation time, easy to rear and cross, and already equipped with established genetic tools for the fine mapping of mutations.

However, many important biological questions are difficult to address with genetic model organisms such as *D. melanogaster* or *C. elegans*. For example (but many more could be chosen), such model organisms do not exhibit complex plastic traits such as caste polyphenism or division of labor within specific colonies which is the case for several social insects such as bees or ants. It thus appears fundamental to develop functional analysis tools in those species that will allow the dissection of the regulatory events of such traits. Another extreme case of phenotypic plasticity is illustrated by aphids. They can indeed respond to specific environmental cues and produce in their offspring alternative and discrete phenotypes. This phenomenon is called polyphenism and occurs during embryogenesis (Le Trionnaire *et al.* 2008; Simpson *et al.* 2011). For example, when aphid population density on a given plant increases, wingless parthenogenetic individuals can perceive crowding signals (increased contacts between individuals or impoverishment of plant nutritive quality) and induce the production in their offspring of winged individuals that can then move to another plant (Sutherland 1969). Another striking example of such phenotypic plasticity is the reproductive polyphenism that aphids can exhibit during their life cycle (Figure 1). They indeed alternate between sexual and parthenogenetic reproduction, depending on the environmental stimuli (reviewed by Le Trionnaire *et al.* 2008). They thus multiply efficiently by parthenogenesis during spring and summer, causing important damages on various crops. At autumn’s arrival, parthenogenetic individuals can perceive the photoperiod shortening and induce in their offspring the production of sexual individuals, males and oviparous females that will mate and lay cold-resistant eggs over winter. Aphids thus offer several advantages to apply a forward genetic strategy because parthenogenetic females can reproduce clonally in favorable environments, without any recombination, such that mothers and daughters are genetically identical. This confers to aphids a unique advantage among insects to store induced mutations at the heterozygous state. They are also able to perform sexual reproduction, which allows setting up genetic crosses between defined stocks. Heterozygous mutations can then be made homozygous by simple brother-sister crosses.

**Figure 1:**
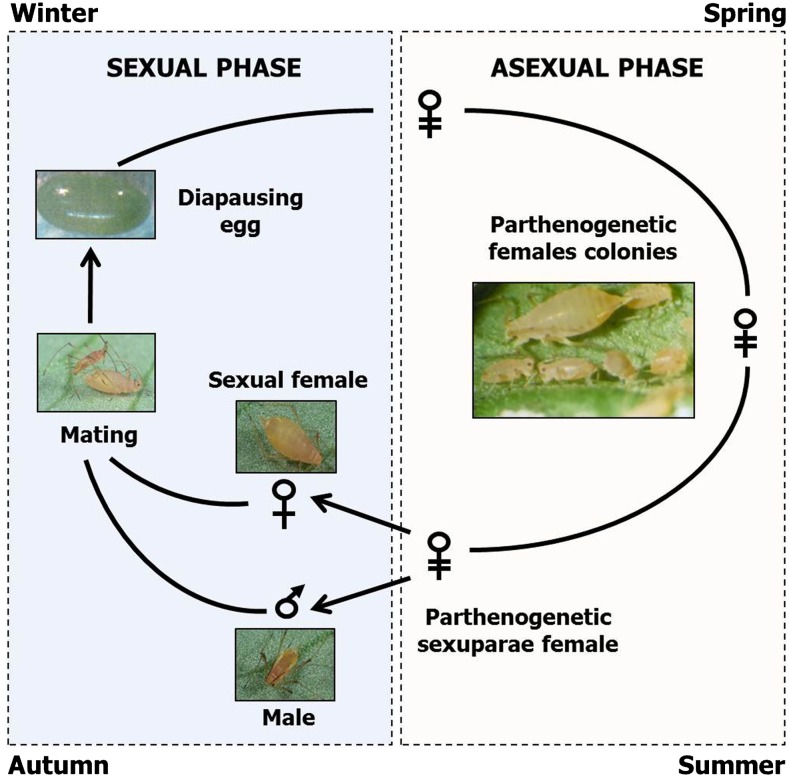
Pea aphid life cycle. During spring and summer, asexual reproduction phase occurs when individuals develop rapidly and efficiently by parthenogenesis. At the end of the summer, parthenogenetic females (also called “sexuparae”) perceive the photoperiod shortening, which induces the production of sexual morphs—males and oviparous females—in their offspring. These individuals will then mate and fertilized females lay cold-resistant eggs that can overcome winter. At the next spring, eggs will hatch and initiate new parthenogenetic colonies.

The pea aphid (*Acyrthosiphon pisum*) has been selected by the aphidologist community to be the reference aphid species to develop genomic resources (Brisson and Stern 2006; Tagu *et al.* 2008). The *A. pisum* genome is now assembled and annotated (International Aphid Genomics Consortium 2010). Functional validation of the annotated genes of the pea aphid is our main motivation in this study to explore the possibility to perform forward genetic screens. In addition, the delicate mapping and molecular identification of EMS-induced DNA lesions is getting more accessible since the decreasing prices of next-generation sequencing technologies have made possible the identification of EMS-induced mutations by whole-genome sequencing. This strategy has been successfully used in *C. elegans*, *D. melanogaster*, and *A. thaliana* (Blumenstiel *et al.* 2009; Doitsidou *et al.* 2010; Uchida *et al.* 2011; Zuryn *et al.* 2010) and opens the way for the identification of DNA lesions also in nonmodel organisms, assuming EMS-based mutational system reveal its efficiency.

Here, we report a protocol of EMS treatment for third stage larvae (L3) of pea aphids to generate random mutations in the genome. We quantified the efficiency of the mutagenesis by estimating the occurrence of mutational events on the X chromosome. As a proof-of-concept, we applied this protocol to a small-scale mutagenesis of parthenogenetic individuals. Among the putative mutant candidates generated, we isolated one stable mutant line for which the photoperiodic response and male morphology were affected.

## Materials and Methods

### Aphid colonies

Two *A. pisum* genotypes were used: LSR1.AC.G1 for which the genome has been sequenced (International Aphid Genomics Consortium 2010) and the genotype P212. This clone is derived from P123, which was collected on pea in the Center of France (Lusignan) in 1999. This clone is known in lab conditions to produce a relatively large proportion of male progeny (approx. 50% and referred as P123 in Simon *et al.* 2011). Aphids are maintained on faba bean (*Vicia fabae*) plants as clonal colonies reared in a controlled environment chamber at 18° with a 16-hr photoperiod. Approximately every 20 d, when colonies are highly developed, three larvae of second or third stage (L2−L3) are isolated and placed on a new healthy plant. The colonies, in these conditions, are all composed of clonal viviparous parthenogenetic females.

### Mutagenesis efficiency assessment

#### Protocol of EMS treatment:

**Figure 2:**
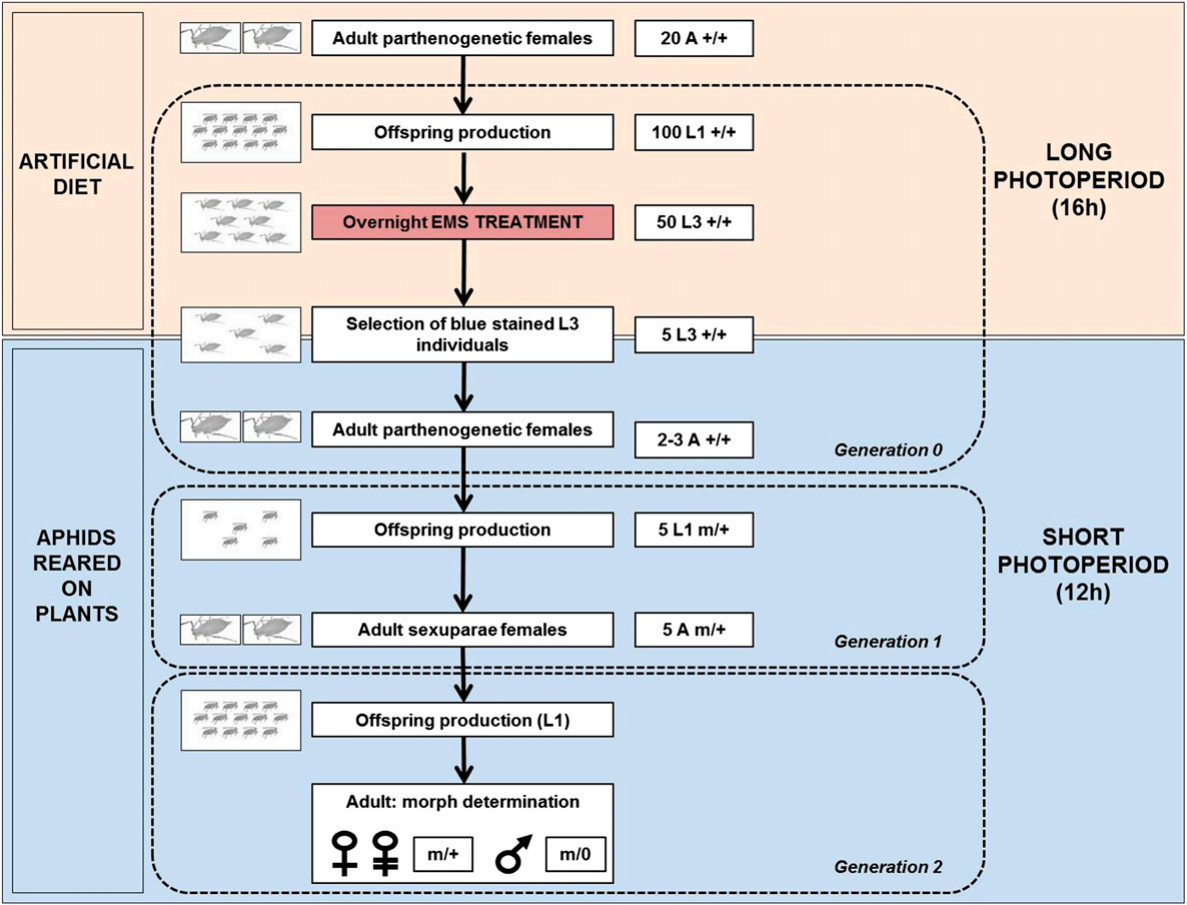
Protocol for pea aphid ethyl methanesulfonate (EMS) treatment and estimation of X-linked mutations. This protocol first allows a correct nutrition of pea aphid larvae on artificial diet under long photoperiod conditions required for the maintenance of colonies by parthenogenesis. Once this habituation step is achieved, aphids are fed onto artificial diet complemented with EMS for 24 hr at L3 stage. They are then moved to plants under a short photoperiod regime (12 hr) that allows the switch of the reproductive mode two generations later. Sexual morphs (oviparous females and males) can then be observed at the third generation. Male proportion within the offspring of EMS treated aphids are used as a read-out to estimate the putative occurrence of X-linked mutations, and thereby the mutagenesis efficiency of EMS treatment. A, adult; L1, first instar larvae; L3, third instar larvae; G0, G1, G2, generation 0, 1, or 2.

EMS treatment was performed on viviparous parthenogenetic females, with each treatment being duplicated at different dates. Viviparous parthenogenetic adult females taken from colonies reared under a 16-hr photoperiod were removed from plants and fed on the Ap3 artificial diet (from 15 to 20 individuals per cell) as described by Febvay *et al.* (1999) with the replacement of the 3.75 mM of β-alanyltyrosine by a supplement of 3.75 mM of phenylalanine. These adults were left 2 d for laying L1 larvae and removed from the artificial diet (Figure 2). Larvae were reared on this artificial diet under a 16-hr photoperiod. This step of adaptation to artificial diet is required to allow a correct nutrition of larvae. Once they reached L3 stage, they were fed on the same artificial diet but complemented with EMS (Sigma-Aldrich M0880). Within each cell, 350 µL of artificial diet was added, complemented with 0 (control), 5, 10, or 15 mM EMS and a droplet of blue staining (Coomassie Blue). From 50 to 75 L3 larvae were installed per cell. Aphids were left overnight in presence of EMS and placed back on a *V. fabae* healthy plant at low density (five individuals/plant). Only aphids displaying a blue staining in their stomach were selected as an indirect proof of their possible feeding on the supplemented medium. For each treatment, 75 to 150 L3 were installed on 15−30 plants. Aphids were then moved to short photoperiod controlled environmental chambers (12hr) to induce the switch of reproductive mode and the associated oviparous females and male production. Mortality of EMS-treated larvae was assessed 72 hr after application.

#### Estimation of the occurrence of X-linked mutations generated by EMS treatment:

In our experimental conditions, it is known that sexual morphs are produced at the third generation (see Le Trionnaire *et al.* 2009). EMS-treated aphids represent the first generation (G0). Progeny assessment is thus performed at the third generation (G2). For this part of the protocol, only P212 clone was used. L3 (G0) individuals treated with different concentrations of EMS (5 mM, 10 mM, or 15 mM) reached adult stage approximately 3−4 d later and began to lay new born L1 (G1) larvae 2 d later. The date of first laying was recorded for each adult individual and treatment. For each living G0 adult, the first five new-born L1 (G1) larvae were selected. All the L1 larvae corresponding to one given treatment were harvested in the same Petri dish, mixed, and subsequently placed onto healthy plants at a density of five larvae per plant until they reached adulthood. These G1 adults are the so-called sexuparae and are parthenogenetic adult female-producing sexual individuals (G2). G1 adults were left to lay down progeny, and to prevent overcrowding on each plant, they were moved to new healthy plants every 2 d while their G2 progeny was kept on the plant. The global offspring of these five adults was then recorded. One replicate thus corresponded to the mixture of the progeny of five individuals. The last step of the experiment consisted in recording the number of individuals produced in the progeny (G2) as well as the type of morph. Morph determination was performed when G2 aphids reached adult stage by using easily recognizable morphologic traits, such as size and color, to help discriminate between males and females and the presence of embryos or eggs, respectively, to distinguish viviparous females from oviparous females (Figure 2). This offspring analysis allowed checking whether EMS treatments could modify the relative proportion of each morph: sexual oviparous females, males, and asexual viviparous females.

#### Statistical analyses:

##### Fecundity and morph distribution:

To estimate EMS effect on fecundity and morph distribution, several replicates were performed for each treatment, a replicate corresponding to the global progeny (G2) produced by five adult individuals (G1) originated from five embryos (G1) contained by the L3 larvae (G0) that fed on the artificial diet complemented or not (control) with EMS. Three independent experiments were thus performed: one where L3 (G0) aphids were treated with 10 mM EMS (12 replicates), another one with 5 mM (18 replicates) and 10 mM (18 replicates), and a third one with 5 mM (18 replicates) and 15 mM (16 replicates). For each of these three experiments, a control corresponding to L3 (G0) aphid fed on an artificial diet without EMS and made of six replicates also was performed. For each replicate, each treatment and each experiment, the total number of individuals produced by the five adults (G1) was recorded as a measure of fecundity, and the morph of each individual was determined to allow a measure of the global morph distribution. To compare the average fecundity between EMS treated and untreated aphids for each of the three experiments and thereby estimate the effect of EMS on progeny size, a one-factor analysis of variance (ANOVA) was performed. To estimate the relative proportion of males and females (sexual and asexual) for EMS-treated and untreated aphids, an independent Khi2-test was performed for each of the three experiments.

##### Mutation rate calculation:

The percentage of observed males was first calculated [N males/(N females + N males)*100] for each treatment (EMS or not). Second, to estimate the mutation-rate (Tx) for each EMS treatment, the following formula was applied: Tx = |ln(%observed males/%expected males)| (Ashburner *et al.* 2005), the percentage of expected males corresponding to the percentage observed within the untreated (control) aphids.

### Mutant generation and maintenance

**Figure 3:**
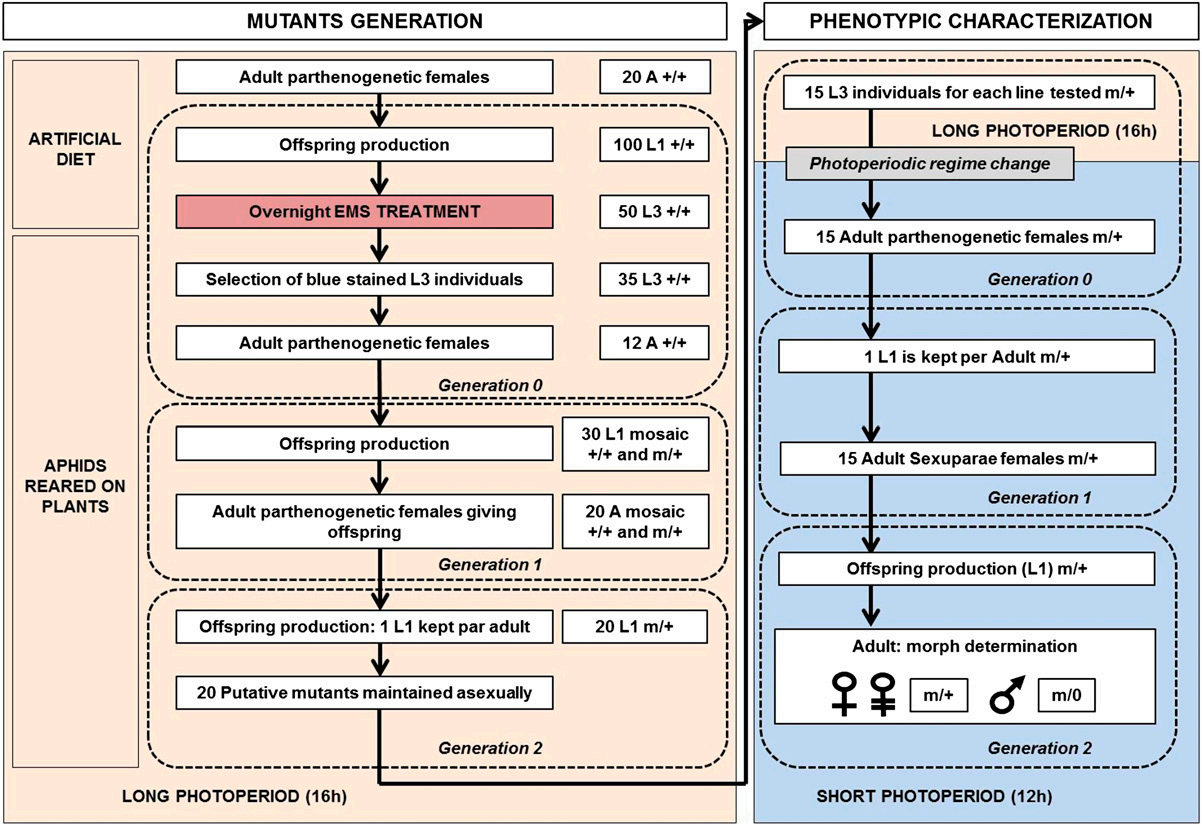
Protocol for pea aphid ethyl methanesulfonate (EMS) mutant generation, maintenance, and phenotypic characterization. The first step of this protocol aims at generating putative mutant individuals and maintaining them as parthenogenetic colonies, which means under long photoperiod conditions (16 hr). Initially fed on artificial diet from L1 stage, L3 aphids were moved to an artificial diet complemented with EMS for 24 hr. Only aphids displaying a blue color (caused by Coomassie Brilliant Blue staining) within their body were conserved and moved to plants. Once adult, a limited number of individuals (12) was conserved, assuming that a large proportion of their embryos were exposed to EMS. Among the progeny of these 12 individuals, around 30 L1 were randomly selected, assuming that the diploid germline of these new-born aphids could have been affected by EMS. Once these individuals reached adulthood, 20 adults were kept and 1 L1 within their progeny was conserved. The 20 L1 conserved thus constituted a stock of putative mutants that could be maintained onto plants by parthenogenesis. The second step of this protocol consisted in the characterization of the photoperiodic response of these putative mutants. To achieve that, L3 individuals picked up from the initial parthenogenetic colonies were moved to a short photoperiod (12 hr) regime. Two generations later (as explained in Figure 2), sexual morphs could be observed. The relative proportion of oviparous females, males and parthenogenetic females within the progeny was then recorded. If a significantly different proportion of males and females as well as some morphological defects were observed repeatedly (three independent biological replicates were performed), the individuals from the EMS-treated aphid stock were considered as putative mutants for their photoperiodic response. A, adult; L1, first-instar larvae; L3, third-instar larvae; G0, G1, G2, generation 0, 1, or 2.

To create and maintain in collection mutant aphids, we developed a dedicated protocol (Figure 3). In this protocol, EMS treatment is still performed on L3 (G0) individuals to target the germline (G2) of their embryos (G1). One hundred L1 (G0) larvae born from 20 parthenogenetic adult females directly fed on an artificial diet were maintained under long photoperiod conditions. Once they reached L3 stage, 50 of them were transferred to an EMS-completed medium at L3 stage. As determined in the experiments above, the concentration of 10 mM was chosen. After 24 hr, blue-stained and surviving individuals (around 35 L3) were moved to *V. fabae* plants until they reached adulthood. Around 12 adult individuals were then randomly kept until they produced an offspring (G1): 30 L1 (G1) offspring were kept under long photoperiod until they reach adulthood. Twenty adults of this G1 were then conserved at that step. These adult G1 produced L1 G2 progeny: only one L1 (G2) individual per adult was then conserved. These 20 L1 individuals were then maintained under a long photoperiod for several generations as independent colonies of “putative mutants.” The fact that aphids need to be reared on plants and that colonies must be changed every 3 wk clearly limits the number of “putative mutants” than can be maintained under collection.

### Phenotypic test

#### Photoperiodic treatment:

Mutant colonies generated and maintained by parthenogenesis were subsequently tested for their photoperiodic response and their ability to produce sexual individuals in their progeny. In this protocol, L3 aphids (G0) from each of the 20 EMS-treated colonies kept in collection (see *Mutant generation and maintenance*) are first isolated from parthenogenetic colonies and transferred to short photoperiod conditions. Once they reach adulthood, one L1 (G1) per adult is conserved and transferred to a new plant. This individual will then reach adult stage 10 d later, and its entire progeny will be isolated (G2) and morphologically characterized. In “wild-type” conditions, the progeny is composed of sexual females, males, and oviparous females born over a period of approximately 15 d. We tested six of the EMS-treated colonies kept in collection for their photoperiodic response. For each of the six EMS-treated colonies as well as for the wild-type colony (P212 clone), the offspring of 10−20 individuals submitted to the photoperiodic treatment and thus corresponding to individual replicates was recorded to allow subsequent statistical analyses.

#### Statistical analysis:

For each of the 6 EMS-treated colonies (putative mutants), we compared the morph distribution following photoperiodic treatment to the wild-type (P212 clone) distribution. The mean number of sexual females, males, and asexual females was recorded and compared between the wild-type and the putative mutant using a one-factor ANOVA. Among the six tested putative mutants, one displayed an altered photoperiodic response. We thus repeated the photoperiodic treatment twice at different periods of the year. The same statistical treatment was applied for each new biological replicate. We then used a Global ANOVA to compare the morph distribution between the mutant and the wild-type within the three biological replicates.

## Results

### Mutagenesis protocol optimization

Our mutagenesis protocol required several steps. First, aphids needed to feed on an artificial diet complemented (or not) with EMS instead of feeding on plants, which requires a habituation step to allow a correct nutrition of larvae. It can be achieved by moving adult females initially fed on plants directly onto the artificial diet to let them lay L1 larvae so that immediately after their birth, these L1 have to feed on this diet. These larvae then show a better nutrition compared with aphids placed on the same diet at later larval stages. Second, the larval stage of EMS treatment needed to be adjusted: L3 stage was selected. It is well known that at this stage germ cells developing into older embryos are already under differentiation (Le Trionnaire *et al.* 2008; Miura *et al.* 2003). Third, it was important to identify aphids that actually fed on the artificial diet complemented with EMS during the 24 hr of treatment to be sure that they properly ingested the mutagen. This selection was made by adding a blue staining to the diet that allowed us to isolate aphids that really ingested the diet and ensured analyzing only the progeny of individuals confronted to the chemical. Even if it allowed the identification of treated individuals, this did not inform on the quantity of EMS ingested by the individuals. In conclusion, this protocol allowed the easy recovery of aphids that ingested EMS, their embryos having a high probability to have been confronted to the mutagen. These treated individuals could then be isolated onto host plants to check for a putative effect of the mutagen on their progeny.

### Aphid viability after EMS ingestion

Among the direct effects of EMS on insect development and fitness, mortality was the most obvious. The percentage of mortality was recorded for two different clones (LSR1 and P212) and three different EMS concentrations (5 mM, 10 mM, and 15 mM) 72 hr after L3 larvae fed on the EMS-complemented diet (Table 1). In all cases, EMS had an effect on the viability of treated aphids, and this effect differed between the two genotypes. Clone LSR1 seemed to be much more sensitive to EMS compared with clone P212, even at low concentrations: 5 mM EMS induced 70% of mortality on LSR1 clone, but only 30% on P212. Although 5 mM had a moderate effect on clone P212, a mortality of 40−50% was recorded for this clone at 10 and 15 mM EMS concentration, respectively. We could thus detect a clone-dependent effect on mortality, even if we couldn’t demonstrate that the two clones had ingested similar quantities of EMS. These results also suggested a dose effect of EMS concentration for a given clone. Individuals treated with higher EMS concentrations (especially 15 mM) were more likely to die compared with aphids treated with lower concentrations (5 or 10 mM). As a comparison, EMS-induced mortality in *D. melanogaster* is around 10% (Bokel 2008), but comparisons are difficult because in both cases, the quantity of ingested EMS is unknown. EMS thus appears to be particularly toxic for aphids. For clone LSR1, the three EMS concentrations induced a loss of fecundity of treated aphids. Dissection of these sterile individuals showed a complete disorganization of their ovaries, with undeveloped ovarioles and no mature follicle (data not shown). It is known that high concentrations of EMS can induce male sterility in *D. melanogaster* (Ashburner *et al.* 2005). This sterility could be the consequence of mitotic defects generated by chromosomal aberrations induced by EMS. In some cases, adult G1 individuals displayed additional abnormal phenotypes such as single aborted wings, which suggested the possibility of somatic effects of EMS treatment. LSR1 viability and fertility was far more reduced after EMS treatment than for clone P212. We could conclude that there was a clear difference in the response to EMS between the two clones. The genetic backgrounds of these two clones are different, but inter-crosses are fertile. The genetic basis of these differences remains unknown.

**Table 1:** Average mortality of two pea aphid clones after feeding with various EMS concentrations

	Experiment 1		Experiment 2		Experiment 3	
Clone LSR1	Control 0 mM	10%	Control 0 mM	10%		
	EMS 5 mM	70%	EMS 10 mM	90%		
			EMS 15 mM	80%		
Clone P212						
	Control 0 mM	10%	Control 0 mM	10%	Control 0 mM	10%
	EMS 10 mM	50%	EMS 5 mM	30%	EMS 5 mM	30%
	EMS 15 mM	40%	EMS 10 mM	30%	EMS 15 mM	50%

Two different pea aphid clones (LSR1 and P212) were fed on artificial diets containing various EMS concentrations (5, 10, or 15 mM). For each treatment, from 50 to 75 L3 individuals were placed on those diets for 24 hr, moved back to healthy plants, and average mortality was recorded 72 hr after EMS treatment. EMS, ethyl methanesulfonate.

### Effect of different EMS concentrations on aphid fecundity and number of putative X-linked lethals

We developed a specific mutagenesis protocol (Figure 2) to test the effect of different EMS concentrations on the global fertility of parthenogenetic aphids. We also estimated the mutation rate generated by the mutagen within their progeny, in that case composed of sexual individuals. Indeed parthenogenetic females (G1) reared under short photoperiods produce in their progeny three types of morphs: sexual oviparous females, males, and a few viviparous asexual females (Lamb and Pointing 1975). Males are hemizygous for the X chromosome (X/0) and thus have only one copy of genes located on this chromosome. Any lethal mutation on the X should then kill males. The number of males produced by an EMS-treated aphid can then be used to estimate the occurrence of X-lethal mutations. The effect of EMS on parthenogenetic females (G1) was assessed by recording the percentage of these three morphs (in G2) within their progeny and compared with untreated females. The total number of each morph was recorded two generations after G0 females were fed with different EMS concentrations. Three independent experiments were performed. We only used the P212 clone in the following experiments as it was more resistant than LSR1 to EMS toxicity.

#### Fecundity:

**Figure 4:**
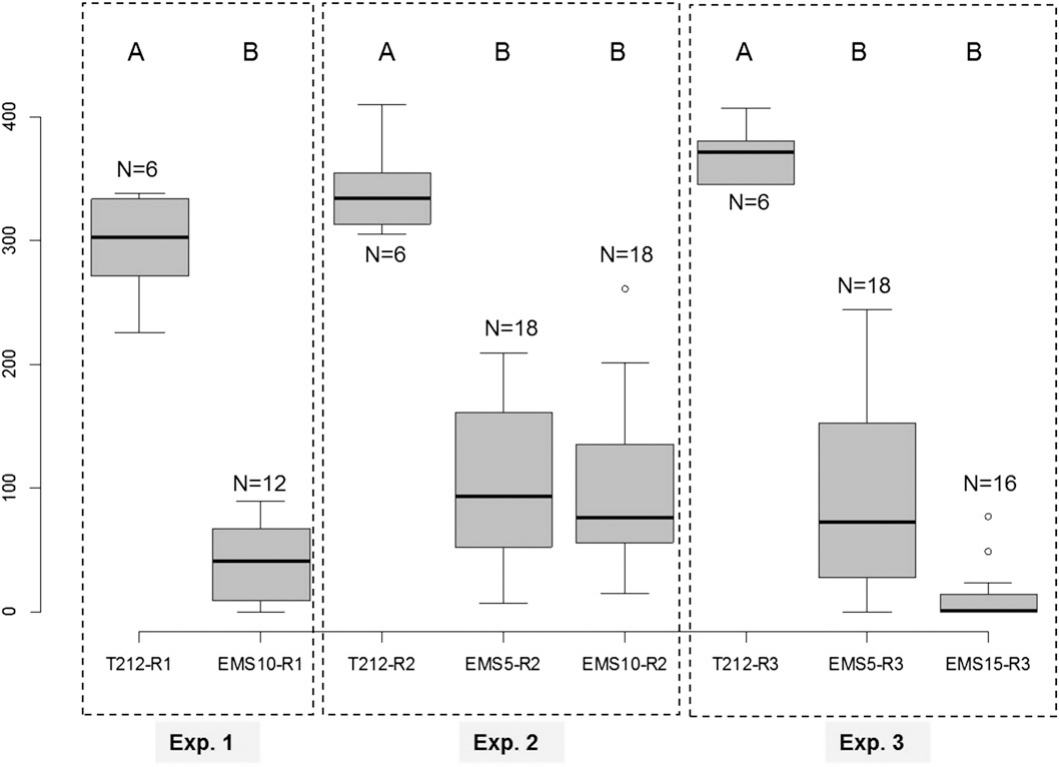
Comparison of average fecundity in the offspring of pea aphids treated with various ethyl methanesulfonate (EMS) concentrations. For each experiment, a one-factor analysis of variance was performed to compare the fecundity of nontreated (T212) *vs.* treated aphids with various EMS concentrations (EMS5 for 5 mM, EMS10 for 10 mM and EMS15 for 15 mM). For each individual experiment and different EMS concentrations, letters (A or B) indicate highly significantly different groups (*P* << 0.0001). Y-axis: total number of individuals produced by treated or nontreated aphids, X-axis: treatment.

For each independent experiment, we first compared the total number of individuals as well as the type of morphs produced within the progeny (G2) of the five first aphids (G1) produced by EMS treated (or not) individuals (G0) (Table 2). One-factor ANOVA analyses performed for each experiment revealed that fecundity was significantly reduced (*P*-value <<0.0001) for aphids treated with 5 mM EMS (average fecundity reduced by 73% and 77% for experiment 2 and 3), 10 mM (87% and 70% for experiment 1 and 2), and 15 mM (97% for experiment 3) compared with control aphids (Figure 4). It thus appeared that treatment with 5 and 10 mM EMS affected fecundity in the same proportions, whereas aphids treated with 15 mM EMS produced nearly no progeny. These results indicated that the germ cells of these adults were affected by EMS treatments when these individuals were still embryos within the EMS-fed parents.

**Table 2:** Morph distribution within the offspring of aphids treated with various EMS concentrations

Experiment	Treatment	Replicates	Fecundity		Morphe Distribution			Male *vs.* Female Distribution		
			Total	Average	OF	M	PF	Average Females	Average Males	Sex Ratio
1	Control 0 mM	6	1776	296.0	640	718	418	176.3	119.7	0.40
	EMS 10 mM	12	480	40.0	194	128	158	29.3	10.7	0.27
2	Control 0mM	6	2052	342.0	721	1171	160	146.8	195.2	0.57
	EMS 5 mM	18	1688	93.8	678	556	454	62.9	30.9	0.33
	EMS 10 mM	18	1803	100.2	713	784	306	56.6	43.6	0.43
3	Control 0 mM	6	2221	370.2	718	1243	260	163.0	207.2	0.56
	EMS 5 mM	18	1568	87.1	670	525	373	57.9	29.2	0.33
	EMS 15 mM	16	184	11.5	63	32	89	9.5	2.0	0.17

After feeding on artificial diet containing various EMS concentrations, aphids were moved to short photoperiod conditions at L3 stage to induce the switch of the reproductive mode (from asexual to sexual reproduction). Once adult, they started to produce their offspring and five new-born larvae were kept per treated individual. Once these larvae reached adulthood, morph distribution (sexual or asexual females and males) as well as the global fecundity of their offspring was recorded. One replicate thus corresponds to the combined offspring of five individuals. To estimate the sex ratio, the average of produced females (sexual and asexual) and males was also calculated. EMS, ethyl methanesulfonate; OF, oviparous female; M, males; PF, parthenogenetic females.

#### X-linked lethals:

**Figure 5:**
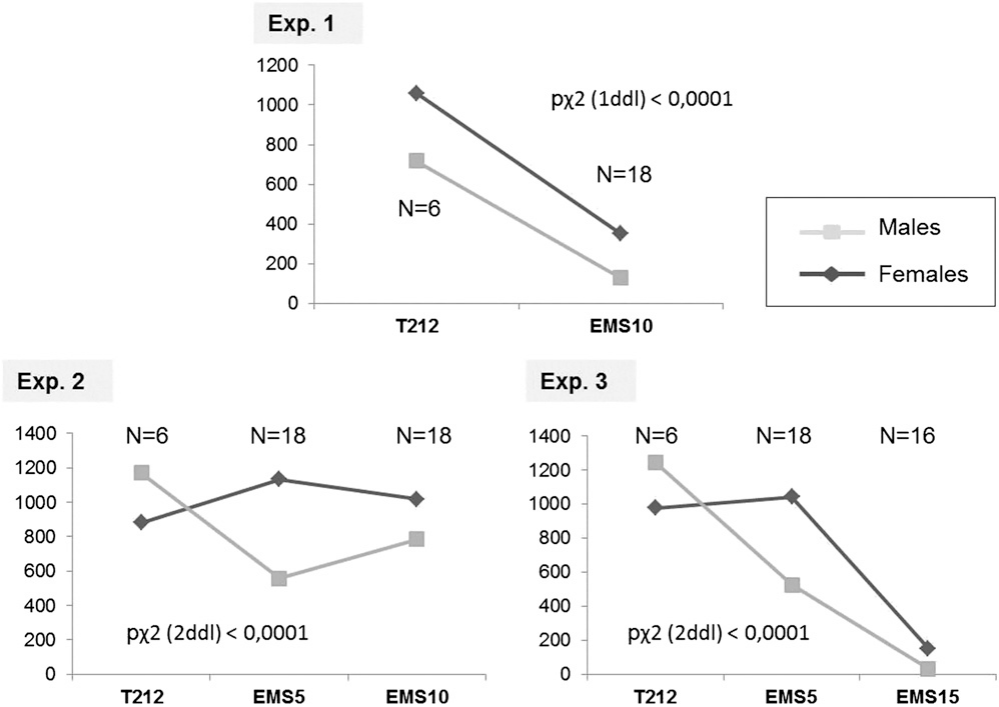
Analysis of the male and female proportion within the offspring of ethyl methanesulfonate (EMS)-treated aphids. The number of females (sexual and asexual) and males produced by EMS-treated aphids was recorded (Y axis) for each experiment and for each EMS concentration (T212 for untreated aphids, EMS5 for 5 mM, EMS10 for 10 mM, and EMS15 for 15 mM). To estimate the relative proportion of males and females for each experiment, an independent χ^2^ test was performed. Significance of the test is indicated on the different graphs. Y-axis: total number of individuals (males or females) produced by treated or non-treated aphids, X-axis: treatment.

As expected for the P212 *A. pisum* clone, the three types of morphs were produced after feeding on an EMS-free artificial diet. Table 2 shows the average number of females (sexual and asexual) and males recorded in the progeny of EMS-fed aphids obtained at different concentrations and for the three different experiments. For the different EMS concentrations tested, it appeared that the average number of males was reduced within the progeny of treated aphids. We also calculated sex ratios [N males / (N females + N males)] for each experimental condition. The sex ratio decreased from 0.4 in the control to 0.27 for aphids treated with 10 mM concentration in the first experiment. In the second one, the ratio was 0.57 in the control and 0.43 for aphids treated with 10 mM EMS and 0.33 for the individuals treated with 5 mM EMS. In the third experiment, sex ratio decreased from 0.56 in the control to 0.33 and 0.17 for aphids, respectively, treated with 5 mM and 15 mM. We then performed independent χ^2^ test to compare the relative proportion of males and females within the progeny of control and EMS-fed aphids (Figure 5). This statistical analysis revealed that these proportions were significantly different between the control and EMS-treated aphids, for the three concentrations tested, with a high level of significance (*P* << 0.0001). We concluded that EMS was efficient to generate X-linked lethals because we could observe a reduced proportion of males and an increased proportion of females in the offspring of EMS-treated individuals.

In *D. melanogaster*, it is assumed that the number of lethal hits follows a Poisson distribution (Bokel 2008). The likelihood for not receiving a lethal mutation, p(0), is thus a function of the average number λ of lethal mutation per X chromosome. p(0) = f = e^−λ^, which can therefore be calculated as λ = −ln (f). f is the ratio (% of observed males after EMS / % of expected males with no EMS). We assumed that the distribution of mutations followed the same rule in the pea aphid. The ratio was estimated for the three different experiments and EMS concentrations (Table 3). The mutagenesis rate was between 0.3 and 0.5 for the 5 mM and 10 mM dose, and 1.17 for 15 mM. These results indicated that there may be a threshold effect between 10 and 15 mM EMS. Alternatively, the high mortality and the low fertility rates found at 15 mM led to a great reduction of the size of the progeny. These results were in the same range as those found in *D. melanogaster* for which 5 mM, 10 mM, and 15 mM EMS treatments induce 0.2, 0.3, and 0.5 sex-linked lethals, respectively (Huang and Barker 1976). Because EMS was ingested by aphids from an artificial diet, it was not possible to know the concentration at which EMS reached the targeted germ cells. This limitation is, however, the same for EMS treatments of *D. melanogaster* or *C. elegans*. Nevertheless, the selection of aphids stained by the Coomassie Brilliant Blue allowed following aphids that ingested EMS. It thus appeared that 5 or 10 mM concentrations of EMS were reasonable to ensure the effective generation of mutants within the progeny of treated aphids.

**Table 3:** Mutation rate calculated for aphid individuals treated with various EMS concentrations

Experiment	2	3	1	2	3
EMS concentration, mM	5	5	10	10	15
% Expected males	57.1	56	40.4	57.1	56
% Observed males	32.9	33.5	26.7	43.5	17.4
Tx	0.54	0.51	0.42	0.28	1.17

The mutation rate is calculated using the following formula: Tx = |ln(%observed males/%expected males)|. The percentage of expected males corresponds to the % of males observed in the control (untreated aphids) for each independent experiment. EMS, ethyl methanesulfonate;

### Mutant generation

**Figure 6:**
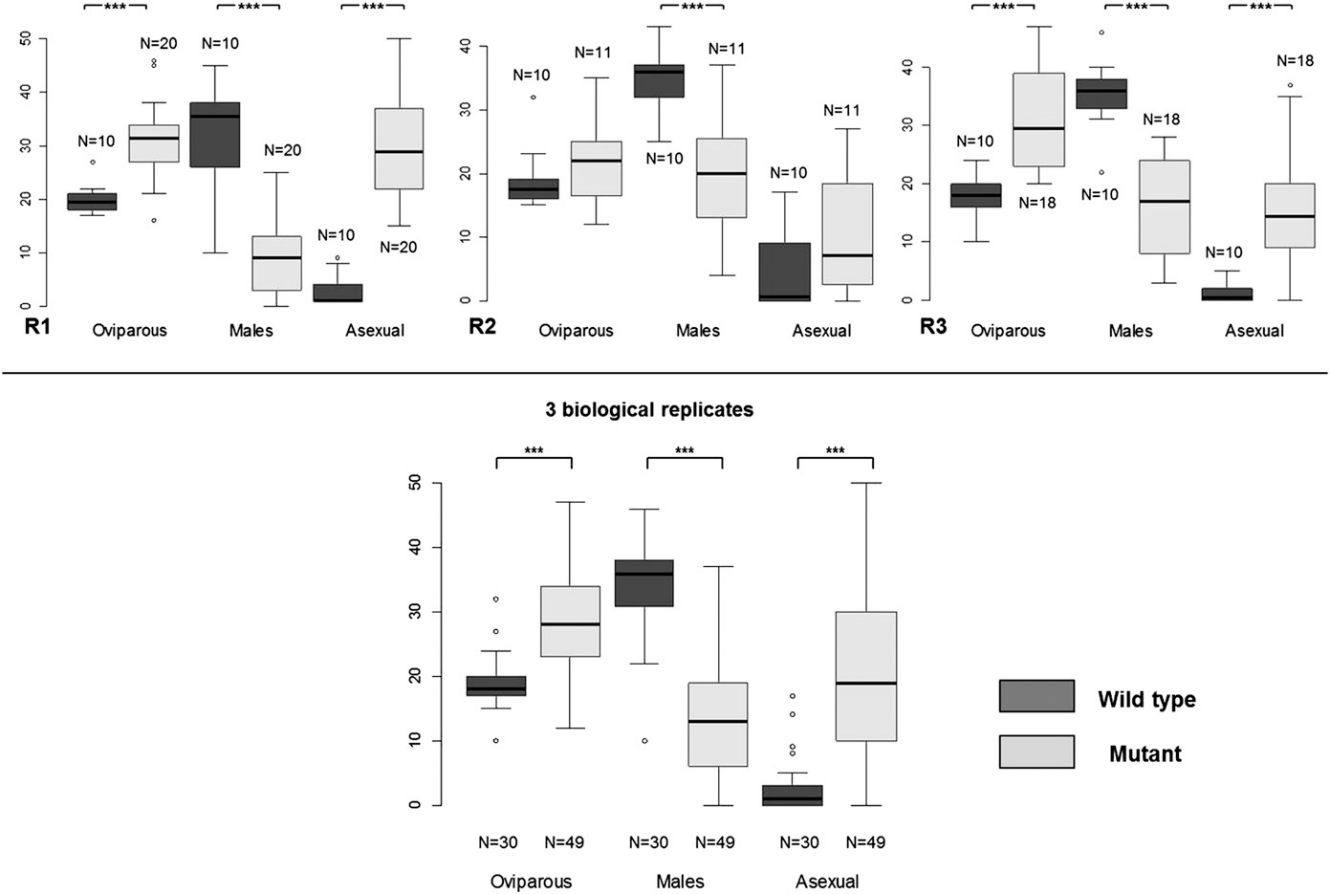
Phenotypic characterization of one ethyl methanesulfonate (EMS) pea aphid mutant. Several putative EMS mutants were generated, maintained by parthenogenesis, and characterized for their photoperiodic response. Among them, one line displayed an altered response, in the sense that it showed a modified proportion of sexual females, males and asexual females in its progeny. For each biological replicate (R1, R2, and R3), the mean number of asexual females, males and oviparous females was compared between the wild-type and the mutant using a one-factor analysis of variance. A global analysis of variance was then performed to compare the average number of each morph produced by the wild-type and the mutant between the 3 biological replicates. Three stars (***) indicate a highly significant difference (*P* < 0.0001) in the average number of a given morph between the wild-type and the mutant. Y-axis: average number of oviparous females, males and asexual females produced by wild-type and mutant aphids, X-axis: type of morph.

**Figure 7:**
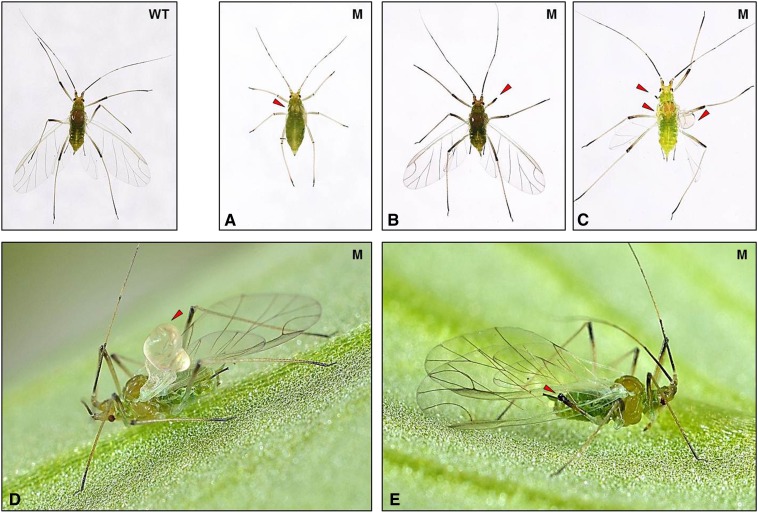
Morphologic defects observed in males produced by the ethyl methanesulfonate (EMS) mutant. Within the different “putative mutants” tested for their photoperiodic response, one showed an altered and reproducible production of males (see Figure 6). In addition, for each independent biological replicate, we could observe within the male population a few individuals displaying obvious morphological defects, which was not the case within the offspring of the control population. These defects corresponded to legs abnormalities such as missing legs segments (b, c, e) and wing defects, ranging from missing wing buds (a) or complete wing parts (c) and in some cases the appearance of stunted wing (c,d). WT, wild type; M, mutant.

Successful optimization of the EMS treatment protocol encouraged us to perform a small-scale mutagenesis in aphids. We thus developed a second protocol to generate and maintain by parthenogenesis some putative EMS-induced mutants (Figure 3).We generated 20 putative mutant lines that were maintained alive and stable by parthenogenesis. This protocol is heavy and time-consuming so that we decided to conserve a manageable number of putative mutant lines. We tested six of these lines for their photoperiodic response (Figure 3) by recording the proportion of sexual females, males, and asexual females within the offspring of the six putative mutants. We used a one-factor ANOVA to compare morph distribution between the wild-type population and the six putative mutants. We found one line showing a morph distribution in its progeny which was statistically different from the wild type. This corresponded to the Replicate 1, where the mean male number within the progeny dropped from 32 to 8.8, whereas sexual and asexual female mean numbers increased from 20 to 31 and from 3 to 29, respectively (Figure 6). We repeated the entire experiment for this line twice at two independent periods of the year and could still observe the same trend, although it was less pronounced in the second biological replicate where only the mean male number was significantly different between the wild-type and the mutant line. We then performed a global ANOVA to combine the data of the three independent experiments, which revealed that the mean number of sexual and asexual females significantly increased (*P* < 0.0001) from 19 to 29 and from 3 to 20, respectively, whereas mean male number significantly decreased (*P* < 0.0001) from 34 to 13.7. These analyses allowed us to conclude that this mutant line showed an altered photoperiodic response with a significantly lower number of males produced. More importantly, this phenotype was detectable over multiple clonal generations, indicating that this mutation was stable and could be transmitted through the germline. In addition to defects in the photoperiodic response, we could observe within the male population of the mutant progeny a few individuals (five or six for each replicate) displaying obvious morphological defects, such as stunted or aborted wings and shortened legs (Figure 7), which could never be seen in the wild-type population. These observations suggested that this mutant line was probably affected in its ability to produce male individuals. We could thus conclude that this protocol allowed us to generate the first EMS-induced aphid mutant, defective for its capacity to produce a balanced ratio of males and females in response to photoperiod shortening and for male morphological development.

## Discussion

This study describes for the first time a protocol of mutagenesis on an aphid genome using EMS. We established a method to deliver a chemical mutagen to pea aphids by feeding these insects on an artificial diet. Our results show that the estimated rate of sex-linked lethal mutations induced by EMS was similar in aphids and in *D. melanogaster* at low concentrations (Ashburner *et al.* 2005). However, EMS was far more toxic for aphid viability and sterility than for drosophila. We found that the degree of EMS toxicity seemed to depend on the genetic background of the aphid clone. The most commonly used concentration of EMS in *D. melanogaster* mutagenesis is 25 mM, which induces a mutation rate of about one lethal hit per X chromosome. At 10 mM, the mutation rate was around 0.4 in the pea aphid and 0.5 in drosophila, which indicates that twice as many chromosomes need to be scored to recover the same number of mutations (Ashburner *et al.* 2005). Our results showed that increasing the concentration of EMS would increase the mutation rate in aphids, but would also greatly reduce the viability and sterility of clones P212 and LSR1. Thus, it could be useful to identify a clone, which would be more resistant to EMS to increase the concentration of EMS in our protocol. Greater concentrations of EMS would require to mutagenize many more adults G0 and to handle many more G1parthenogenetic individuals, whereas lesser concentrations would require the screening of many more G2 individuals. We can thus propose that 10 mM of EMS is a good compromise for the pea aphid. Alternatively, it may also be interesting to test other chemical mutagens such as methyl methanesulfonate or *N*-ethyl-*N*-nitrosourea.

Building on this proof-of-concept, the following step was to find a way to generate and maintain under collection some putative mutant lines that could then be tested for a phenotype of interest. The main issue in designing such a genetic screen is whether one is looking for recessive or dominant mutations. Recessive mutations are more likely to represent a loss-of-function allele of a gene, but they are only visible at the homozygous state. It requires two steps of crosses going through two generations of sexual individuals (a classic F3 screen in *D. melanogaster*, but without markers and balancer chromosomes). It is a fair amount of work for one specific aphid clone (nearly a year), but it becomes a daunting task when one has to screen hundreds of mutagenized lines. In contrast, dominant mutations are visible directly in the F1 progeny. On the down side, those alleles are often more difficult to interpret as they can be loss- or gain-of-function alleles, or even neo- and antimorphic alleles. It can then be hard to relate the observed phenotype with the endogenous function of the mutated gene. The aphids offer, however, the immense advantage to perform F1 screens on parthenogenetic females. Using our protocol, it was possible to score for phenotypes two generations after EMS feeding of parthenogenetic females raised in long photoperiods. Each isolated and interesting mutant could then be propagated as a clone, as parthenogenetic females do not recombine. It thus becomes a real prospect to establish collections of mutant aphid bearing dominant mutations. We thus developed a protocol allowing the exposure of L3 larvae (G0) to 10 mM EMS, hoping for the mutagen to target the germline (G2) of embryos (G1) contained within the treated individual (G0). Aphids indeed represent a complex biological system, mainly due to the embedment of generations within parthenogenetic individuals. Aphids thus contain embryos that themselves contain already developed germlines. Stable mutation requires targeting germline cells so that for a given individual treated with EMS (G0), only aphids from the third generation (G2) could contain stable mutants. Taking into account this particularity of the aphid model, we finally managed to conserve 20 putative mutant lines and actually tested six for their photoperiodic response. Among these six lines, tested individuals from one line showed a repeatable and reduced proportion of males and an increased proportion of females within their offspring. Some of the males produced by this line could also display morphologic defects, which led us to the hypothesis that such mutation could be a recessive mutation on the X-chromosome or a dominant mutation on an autosome, in a gene specifically required for male development. Furthermore, this experiment (Figure 3) differs from the first mutagenesis experiment (Figure 2), where random and unrelated mutations were analyzed at the population level for male lethality. In this second experiment, a single line showed lower number of males than expected, and some of the surviving males showed developmental defects. However, these different phenotypes could be due to one or several mutations, and although only males are affected, it doesn’t mean that this/these mutation(s) are the X chromosome.

The mapping and molecular identification of DNA lesions induced by EMS remains to be made to identify the gene(s) responsible for the observed phenotype. Such mapping step remains a clear bottleneck of this forward genetic strategy in any model organisms. However, the price of whole-genome sequencing has greatly decreased. It then becomes possible to directly sequence the mutated genomes and identify differences with a reference genome. This approach has been successful in *C. elegans*, *D. melanogaster*, and *A. thaliana* (Blumenstiel *et al.* 2009; Doitsidou *et al.* 2010; Uchida *et al.* 2011; Zuryn *et al.* 2010). Interestingly, these studies also revealed that EMS induces many more mutations than 1 lethal hit per chromosome arm. It was found that a 25−50 mM EMS concentration induces on average one mutation every 150−300 kb, which means several hundreds of mutations per genome. Only 10% of those fail into open-reading-frames and lead to gene inactivation. Nevertheless, sequencing will not help to distinguish between the 10 or so remaining candidate genes with base pair changes, and a mapping step is still very advisable. The most cost-effective method to date is to combine in one step the sequencing of the whole genome and the mapping of the mutation by single-nucleotide polymorphisms (Doitsidou *et al.* 2010). This method only requires the generation of approximately 40−50 aphid lines bearing the mutation of interest and that are recombinant between the mutagenized line and a polymorphic strain. The genomes of all these recombinants can then be pooled and sequenced only once. A decrease in the density of polymorphic markers indicates the region where the phenotype-causing mutation is localized. This strategy has the additional advantage to produce mutant lines in which the genetic background has been cleaned of second-site mutations.

We hope our protocol will help validating functionally the increasing and exciting amount of novel genomic data available for *A. pisum*. The establishment of collections of mutants for various phenotypes should then be shared between labs and benefit the entire community, such as the International Aphid Genomics Consortium.
